# Grand Challenges in Global Mental Health: Integration in Research, Policy, and Practice

**DOI:** 10.1371/journal.pmed.1001434

**Published:** 2013-04-30

**Authors:** Pamela Y. Collins, Thomas R. Insel, Arun Chockalingam, Abdallah Daar, Yvonne T. Maddox

**Affiliations:** 1 Office of the Director, National Institute of Mental Health/NIH, Bethesda, Maryland, United States of America; 2 World Hypertension League and Faculty of Health Sciences, Simon Fraser University, Vancouver, British Columbia, Canada; 3 Grand Challenges Canada and Dalla Lana School of Public Health and Department of Surgery, University of Toronto, Toronto, Ontario, Canada; 4 Stellenbosch Institute for Advanced Study (STIAS), Wallenberg Research Centre at Stellenbosch University, Stellenbosch, South Africa; 5 Office of the Director, Eunice Kennedy Shriver National Institute of Child Health & Human Development/NIH, Bethesda, Maryland, United States of America

## Abstract

In the first article of a five-part series providing a global perspective on integrating mental health, Pamela Collins and colleagues set the scene for why mental health care should be combined with priority programs on maternal and child health, non-communicable diseases, and HIV, and how this might be done.

**Summary Points**

- Mental illnesses frequently co-occur with peripartum conditions, HIV-related disease, and non-communicable diseases. Care for mental disorders should be integrated into primary care and other global health priority programs.
- Integration of care for mental, neurological, and substance use (MNS) disorders should (1) occur through intersectoral collaboration and health system-wide approaches; (2) use evidence-based interventions; (3) be implemented with sensitivity to environmental influences; and (4) attend to prevention and treatment across the life course.
- Integration of care for MNS disorders with care for other conditions can occur through assimilation of activities, policies, or organizational structures at local, national, and global levels.
- Plans for health-related development targets post-2015 should consider the tremendous burden of disability associated with MNS disorders and co-morbid conditions.
- This paper is the first in a series of five articles providing a global perspective on integrating mental health.

*This is one article in a five-part series providing a global perspective on integrating mental health.*

## Introduction

More than a decade ago, the World Health Organization's (WHO) World Health Report 2001 called for the integration of mental health into primary care, acknowledging the burden of mental, neurological, and substance use (MNS) disorders globally; the lack of specialized health care providers to meet treatment needs—especially in low- and middle-income countries (LMICs); and the fact that many people seek care for MNS disorders in primary care [1]. In 2012, the Global Burden of Disease (GBD) Study 2010 confirmed the still urgent need for attention to MNS disorders: over the past 20 years, the disability adjusted life years (DALYs) attributable to MNS disorders rose by 38%, and mental and behavioral disorders account for nearly one quarter of all years lived with a disability [2],[3]. MNS disorders also contribute indirectly to mortality, through suicides and conditions like cirrhosis, which, in certain regions, both rank among leading causes of disease burden [2].

The GBD Study 2010 brought welcome news of reductions in the DALYs for communicable, maternal, neonatal, and nutritional disorders since 1990. This progress is due, in part, to coordinated, global cooperation to meet the Millennium Development Goals (MDGs) and, specifically, to achieve targets set for child survival, maternal health, and combatting HIV/AIDS and malaria by 2015. Crucial for the global public health community, investments in achieving the health-related MDGs catalyzed the development, testing, and implementation of effective health interventions for priority conditions and stimulated the development of packages of care that bundle effective interventions—whether for reduction of maternal or child mortality or for HIV care and treatment. Stakeholders recognize that “synergies in the health system must be pursued” [4], and that these packaged interventions can be delivered most effectively through integrated approaches to care [5].

The need for integrated care that addresses emerging priority conditions, like non-communicable diseases (NCDs), including MNS disorders, is acknowledged less frequently in the global context [6]. As a result of global population growth, aging, and epidemiologic and demographic transitions, NCDs account for more than 60% of deaths worldwide, with disproportionate rates of mortality among populations in LMICs [7]. Significantly, MNS disorders frequently occur throughout the course of many NCDs and infectious diseases, increasing morbidity and mortality [8]–[11]. Consequently, people suffering with co-morbid disorders, such as depression and HIV or post-traumatic stress disorder and coronary heart disease, risk poor outcomes for both disorders. Achieving desired outcomes for priority programs will be difficult without managing MNS disorders.

At a minimum, packages of care for MNS disorders should be parceled with effective interventions in primary care or other priority health delivery platforms. In truth, adequate attention to the public's health requires that this integration also occur in sectors beyond health (e.g., education, justice, welfare, and labor), through collaborative partnerships of government, non-governmental organizations (NGOs), and faith-based organizations, as well as in the implementation of global health and development policy.

## The Grand Challenge of Integrating Care for MNS Disorders with Other Chronic Disease Care

Despite the increasing burden of MNS disorders around the world and their frequent co-morbidities, affected individuals often lack access to mental health care in high-, middle-, and low-income countries [12]. Inadequate investments in mental health care are partially responsible. Costs associated with mental illness are expected to reach US $6 trillion globally over the next 15 years [13]. Co-morbid illness increases costs by around 45% for each person with a mental disorder and another chronic condition [14]. Yet, governments, on average, spend less than US $2 per person per year on mental health care; a 200-fold difference in per capita expenditures exists between high-income- and low-income countries [15]. The funds allocated to mental health care are directed disproportionately to the provision of beds for psychiatric patients in mental hospitals, which receive as much as 67% of monies spent on mental health [15]. The WHO has long advocated for community-based mental health services in all countries, which would permit a diversity of service settings, increase access to care, and utilize community health resources more efficiently [1].

In 2011, in an effort to stimulate solutions to these and other issues, researchers, clinicians, and advocates identified 25 Grand Challenges in Global Mental Health (GCGMH); i.e., “specific barriers that, if removed, would help to solve an important health problem. If successfully implemented, the intervention(s) … would have a high likelihood of feasibility for scaling up and impact” [16]. The GCGMH provide a clear set of priorities that emphasize needs in low-, middle-, and high-income countries. One year later, the United States National Institute of Mental Health (NIMH), in collaboration with the Eunice Kennedy Shriver National Institute of Child Health & Human Development, the Fogarty International Center, and the National Heart, Lung, and Blood Institute, of the National Institutes of Health, convened a group of experts from programs targeting HIV prevention and care, maternal/child health, and NCD care for a workshop focused on one of the Challenges: “Redesign health systems to integrate MNS disorders with other chronic-disease care, and create parity between mental and physical illness in investment into research, training, treatment, and prevention.” The aim of the workshop was to identify the best ways of integrating mental health interventions into existing global health platforms of care in order to reduce the burden of disease and disability associated with mental disorders while simultaneously helping to achieve the desired outcomes for these broader programs. In a series of five articles in *PLOS Medicine*, workshop participants focus on mental health care integration into maternal and child health programs [17], NCD care [18], HIV care and treatment [19], and on competencies and work packages for services in these contexts [20]. This paper serves as the introductory article.

Four broad themes running across the Grand Challenges are consistent with arguments in favor of integration: use system-wide approaches, apply evidence-based interventions, understand environmental influences, and use a life course approach.

### Use System-wide Approaches

Integration lies at the heart of system-wide approaches to care and aims to “create coherence and synergy between various parts of the health care enterprise in order to enhance system efficiency, quality of care, quality of life, and consumer satisfaction, especially for patients with complex and multiple problems” [21]. Every part of a health system (i.e., health services, the health workforce, health information systems, technologies, health financing, as well as leadership and governance) can play a role in the ultimate goal of delivering quality interventions that reduce the burden of MNS disorders in the population [22]. A system-wide approach requires that mental health information systems—e.g., methods for population-level data collection on mental health outcomes or individual case-level data captured in medical records—are integrated into information systems used throughout the health care system [23]. Likewise, procurement of medications for MNS disorders must not be limited to psychiatric hospitals; rather, these medications, as well as psychosocial interventions, should be available for use at secondary and primary care encounters. Additionally, this approach could extend the use of mobile phones for adherence support and treatment protocols for epilepsy, depression, or psychosis, and would ensure that mental health is equitably incorporated into financing and governance activities.

System-wide collaboration must go beyond the health sector. The well-being of the most vulnerable of health system users, whose symptoms due to mental or physical disorders lead to persistent impairments, may be a sensitive indicator of a society's need for integrated care [21]. Full social participation for vulnerable groups requires sustained access to jobs, schools, and other services; this requires cooperation among education, social services, labor, and justice sectors [21],[24].These efforts can be tailored to available resources; in small villages of Rio Negro Province, Argentina, intersectoral collaboration occurs when mental health professionals collaborate with justice officials to intervene in cases of domestic violence or to train and equip local police staff to recognize acute psychiatric problems and to facilitate home-based care [25]. NGOs often facilitate integrated delivery of mental health and social services in community settings [26],[27] (see Box 1).

**Box 1. NGO Responses to Community Mental Health Needs: Integrating Mental Health, Social, and Economic Support**

The *BasicNeeds Mental Health and Development Program* bridges sectors within and outside the health system in order to integrate health and social interventions for people with epilepsy and mental illnesses. The Nepal program, one of several international sites, operates through a local nongovernmental organization (NGO) with expertise in community-based rehabilitation and livelihoods support. In order to identify affected people, raise community awareness of mental illness, ensure access to treatment, and support employment, collaboration occurs among community members, district hospital staff, NGO staff, families, and affected individuals. In this setting, community-based workers facilitate access to mental health services, monitor treatment through home visits, and support livelihood activities [26].

*Services for People with Disabilitie*s is a community mental health program in Abuja, Nigeria that provides services for people with psychosis and epilepsy. Established by CBM– an international nongovernmental organization that supports programs for people with disabilities in low-income countries–the Catholic Archdiocese of Abuja runs and partially funds the program. The program also delivers community-based rehabilitation services for people with physical disabilities; activities include interventions to support economic integration and social inclusion. Field workers visit local households to identify potential clients, consulting with village chiefs, parish priests, and other local informants. The program raises awareness of its services through public meetings and discussions with other NGOs and with government officials. A community psychiatric nurse delivers care in the home on a monthly basis [27].

### Use Evidence-based Interventions

Over the past decade, the potential for delivery of evidence-based packages of care for mental disorders has grown globally. Interventions that effectively treat mental disorders in high-income countries increasingly demonstrate efficacy in LMICs in varied settings with different kinds of health care providers [28]–[31]. The research generating this evidence base has contributed to the development of packages of care for the whole spectrum of MNS disorders and the workforce capacity-building tools needed to implement them [32],[33].

The WHO Mental Health Gap Action Programme (mhGAP), through its mhGAP Intervention Guide, outlines interventions focused on depression, psychosis, bipolar disorder, epilepsy, developmental and behavioral disorders in children and adolescents, dementia, alcohol use disorders, drug use disorders, and self-harm/suicide [32]. Developed for non-specialists, the guide should facilitate more efficient delivery of services, as well as training and supervision of general practitioners in the use of clinical protocols, algorithms, and decision support tools. Moreover, these interventions can be delivered in the most relevant treatment setting depending upon the unique circumstances of the health system—e.g., distribution of MNS disorders in the population, availability of providers, demand for services, etc.—be it primary care or maternal and child care.

### Understand Environmental Influences

Every attempt to integrate services or strengthen components of a health system occurs in a particular sociocultural, political, and health system environment with its unique configuration of risk factors across the population. Among these factors, epidemics, war, criminal violence, migration, discrimination, and poverty shape the health outcomes and life chances daily for billions worldwide. These conditions disrupt opportunities for maintenance of mental health and often increase risk for mental disorders, influencing the distribution of distress and illness in local settings [24].

In every setting, addressing mental health care needs requires consideration of how individuals understand their symptoms of mental illness or distress and, at the health system level, the ability to utilize available infrastructure and human resources. In low-income settings post-disaster, this may entail building on the resources of existing NGOs to mobilize mental health services in collaboration with local government [34]. In fragile states, during and following prolonged conflict, opportunities to reconstruct a health system can also provide prospects for integrating mental health services into routine health care services, as in the case of Afghanistan [35]. Context should guide decision makers' assessment of whether supporting mental health services through priority programs or through primary care services provides the best opportunities for optimal, sustainable disease prevention and management.

### Use a Life Course Approach

Physical and social exposures at every stage of life influence risk for disease across the life cycle [36]. Integrated care lends itself to holistic management of exposure to risk, the delivery of preventive interventions, and the treatment of symptomatic disease across the lifespan. Poor maternal nutritional status affects in utero neurodevelopment and can significantly increase the risk for major mental disorders that may manifest in childhood or young adulthood and persist across the life cycle [37]. Maternal depression increases the likelihood of preterm birth and low birth weight in some populations [38]; low birth-weight infants also carry greater risk for developing NCDs such as diabetes, hypertension, and heart disease in adulthood [39]. An infant with a depressed mother may be at greater risk for underweight and stunting and for cognitive as well as harmful metabolic disturbances later in life [38],[39].

Symptoms for most mental disorders express themselves in adolescence. Preventive and early interventions that precede and continue into adolescence will have better reach through integrated services—particularly those occurring in schools and community settings outside of the health system. At older ages, the interaction of risks continues: accumulating co-morbid physical conditions increase risk for poor mental health as well as other NCDs [40]. Midlife and late life exposure to depressive symptoms, in turn, can increase risk for dementia [41], whereas continued learning in older age helps prevent cognitive decline.

### Operationalizing Integration

Grépin and Reich provide a useful framework for conceptualizing integration—i.e., what is integrated into what—that links these themes to actions [42]. The framework defines *domains* of integration: what is being integrated (activities, policy, organizational structures); level of integration, or where integration is occurring (local, national, or global); and degree of integration, or how integration is occurring (e.g., coordination of communication and information exchange, collaboration, consolidation of programs or efforts). Table 1 proposes relevant integration actions for mental health services.

**Table 1:** Operationalizing integration: Examples of activity, policy, and organizational integration that link MNS research and care to other health-related programs.

Domain	Level		
	Global	National	Local
Activity	Collaboration of Grand Challenges Canada's “Saving Brains” and “Global Mental Health” research and innovation programs	Integrating the WHO mhGAP Intervention Guide into national or regional primary care training	Implementation of the collaborative care model for management of depression and NCDs in primary care clinics
Policy	Integration of goals relevant to mental health into the post-2015 targets	Coordinated guidelines for intersectoral collaboration on rehabilitation services for people with MNS disorders	NGO advocacy for parity for mental health and NCD expenditures in local and regional government budgets
Organizational structure	Formation of the Global Alliance for Chronic Disease, a global consortium, the membership of which represents research funding organizations that support research on NCDs, including mental health	Incorporation of mental health outcomes and determinants into routine national surveillance programs	Consolidation of community health worker programs to integrate training and screening for MNS disorders

Note: Adapted from Grépin and Reich [42]. *Domain* indicates what is being integrated; *Level* indicates where integration is occurring. Text indicates the form of collaboration occurring.

## Parity Post-2015: From Research to Practice and Policy

The GCGMH also calls for parity “between mental and physical illness in investment into research, training, treatment and prevention”. A flexible, dynamic relationship between research and service delivery enables innovative “game changing” in global public health [43]. Interventions for treatment and care of HIV were informed by rapid monitoring and evaluation; shifts in regimens; and a global, coordinated uptake of data-driven findings. This ability to rapidly study and coordinate a response to MNS disorders does not exist. The availability of research on these disorders varies dramatically across countries, in part due to the distribution of research investments, workforce, and capacity.

The European Union, the United States, China, Japan, and Russia account for 35% of the world's population, but 75% of its researchers [44]. In the case of mental health, fewer than one researcher per 1 million persons studies these issues in LMICs [45]. The disparities are more evident among specific study populations: 90% of randomized controlled trials of mental health interventions for children have been conducted in high-income countries, but 90% of the world's children live in LMICs [46]. Recent investments in mental health services research in LMICs, however, are contributing to a change in the landscape [47]–[50], although more is needed. Over the next 5 to 10 years, a growing body of evidence on the effectiveness of task-shifting interventions, scaling up of mental health service delivery, and integration of mental health into chronic disease care in LMICs will be available [51].

Dissemination of study findings to decision makers, practitioners, managers, and advocates will be crucial to ensuring uptake of innovations. Research partnerships that include mental health service users, government officials, and knowledgeable stakeholders can facilitate dissemination to desired audiences. NIMH has required that investigators utilize these kinds of collaboration in its global mental health research funding announcements [47]–[49]. Ideally, such collaborations would also facilitate bidirectional communication that informs researchers and policymakers of innovations and advocacy ongoing in community settings while simultaneously harnessing the combined resources of researchers, activists, civil society, and other stakeholders to generate and disseminate knowledge to meet the Grand Challenges.

The existing evidence base, however, demonstrates that parity for MNS disorders in policy development is particularly relevant as plans for health-related development targets for the agenda after 2015 progress. As universal health coverage emerges as a feasible option, equitable coverage for MNS disorders must be clearly articulated so that targets and metrics take into account the significant disability associated with these conditions in every region of the world [3].

## The Time Is Right for Integration

The fast approaching deadline for achieving the MDGs, the articulation of health-related targets for the post-2015 development agenda, the call for an AIDS-free generation, the imperative of equitable health care for all individuals (regardless of co-morbid conditions), and the requisite health systems strengthening activities that enable the meeting of these goals make this an ideal time to integrate mental health care services into priority global health programs. There are six other reasons.

1. Just as progress toward the MDGs is in part attributable to shared global goals and vision, a clear agenda, and measurable targets [52], similar progress in the mental health arena is expected. The global mental health community has come to consensus regarding priorities. Moreover, the World Health Assembly's adoption of Resolution WHA65.4—*Global Burden of Mental Disorders and the Need for a Comprehensive, Coordinated Response from Health and Social Sectors at the Country Level*, and the comprehensive 2013–2020 Global Mental Health Action Plan that has emerged, will encourage coordinated global expansion of mental health service delivery. The Plan is timely because the field is already prepared to move selected evidence-based interventions forward for scale-up.

2. Donors are actively directing funding toward reducing morbidity and mortality associated with priority conditions identified by the MDGs [53]. Success in achieving these goals depends, in part, upon adherence to care, the ability to reduce risky health behaviors, the capability of caregivers—especially mothers—to attend to the nutritional and preventive health needs of their children, and maintaining adequate financial resources in households—all of which are actions severely curtailed by mental illnesses [54]. The reality, however, is that some targets will likely not be met by 2015. Now is the time for innovations that will improve continued efforts to reduce child mortality, end HIV transmission, and reduce maternal mortality—and attention to mental health must be part of this effort.

3. As those committed to improving health and development discuss promotion of universal health coverage as a potential development target post-2015, MNS disorders must be integrated equitably among the health conditions considered.

4. The United States Agency for International Development (USAID) and like donors not only emphasize the importance of strengthening health systems, but also are exploring how to leverage the infrastructure gained through investments in HIV/AIDS, maternal and child health, family planning, and other key programs to achieve this goal [53]. Organizations such as Partners in Health and global development platforms such as the Millennium Villages Project are testing models of care that integrate mental health into priority programs [55].

5. The effects of the global economic crisis underscore the need for greater efficiency in health programs while maintaining maximum effectiveness to ensure sustained productivity among populations—an aim of care integration.

6. The evidence demonstrating that there truly is “no health without mental health” continues to grow, and the links between HIV and depression, cardiovascular disease and anxiety disorders, diabetes and depression—as well as other conditions—suggest that the best outcomes for these disorders require care that attends to all of them [6].

The sixth goal of the GCGMH is “to transform health system and policy responses to MNS disorders worldwide”. Despite our worldwide focus, the authors acknowledge their current affiliations with institutions in high-income countries and recognize the importance of perspectives from low-, middle-, and high-income countries. In the five-part series providing a global perspective on integrating mental health, we aim to help health care providers, donors, and decision makers understand the importance of including mental health care in global health programs, identify entry points for integration, select interventions to be introduced into existing health services, and take steps toward action.

## Abbreviations

DALY: disability adjusted life year
GBD: global burden of disease
GCGMH: Grand Challenges in Global Mental Health
LMIC: low- and middle-income country
MNS: mental, neurological, and substance use
mhGAP: Mental Health Gap Action Programme
MDG: Millennium Development Goal
NCD: non-communicable disease
NIMH: National Institute of Mental Health
NGO: non-governmental organization
WHO: World Health Organization
